# Chromosome-level genome assembly of the ornamental plant *Alcea rosea*

**DOI:** 10.1038/s41597-025-05473-z

**Published:** 2025-07-04

**Authors:** Xi Chen, Xiu Li, Shengwen Tang, Jiao Ma, Zhangshun Zhu, Fangwen Li, Xiaoqing Shi

**Affiliations:** Chengdu Botanical Garden, Chengdu, Sichuan China

**Keywords:** Genome duplication, Plant evolution, Genome assembly algorithms

## Abstract

*Alcea rosea*, a member of the Malvaceae family, is celebrated for its rich floral palette and global horticultural significance. Here, we present a high-quality reference genome for *A. rosea*, achieving a genome assembly size of 1.01 Gbp, with a Contig N50 length of 36.61 Mbp. The genome sequence was successfully mapped to 21 chromosomes, and the scaffold N50 length reached 52.57 Mbp, with a scaffold genome completeness of 99.6%. A total of 565.84 Mbp (comprising 56% of the genome) of repetitive sequences were identified, with transposable elements being predominant, particularly long terminal repeat (LTR) elements, which accounted for 48.44% of the genome. 51,436 genes were annotated. Among these predicted genes, the average gene length and coding sequence (CDS) length were 2739.92 bp and 1242.54 bp, respectively.

## Background & Summary

**Fig. 1:**
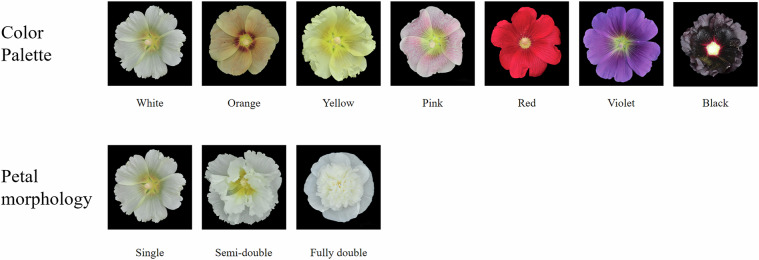
The diverse color and petal types of *A. rosea*.

The Malvaceae family encompasses both economically vital crops and ornamental species. Economically, it includes globally significant crops such as cotton (*Gossypium* spp.) for natural fiber production and cacao (*Theobroma cacao*) for chocolate manufacturing^1–3^. Ornamentally, the genus *Hibiscus* (e.g., *H. syriacus*, *H. mutabilis*) has been extensively studied for its horticultural value. As a member of the Malvaceae, hollyhock (*A. rosea*) holds unique dual significance. Historically cultivated in China for over two millennia, it was documented in ancient pharmacopeias like Compendium of Materia Medica for its edible and medicinal properties. Modern studies have identified bioactive compounds in hollyhock, including flavonoids and polysaccharides with demonstrated anti-inflammatory and antimicrobial activities^4–7^. Beyond its traditional uses, hollyhock exhibits exceptional floral diversity, with petal colors ranging from white to black and intricate variegation patterns, making it an ideal system for studying pigmentation genetics (Fig. 1).

Prior genomic efforts within the Malvaceae family have predominantly focused on economically pivotal species. The genus *Gossypium* (cotton) boasts multiple high-quality genomes, including the diploid *G. raimondii* and tetraploid *G. hirsutum*, which revolutionized fiber development studies^8,9^. Similarly, the cacao genome and its re-sequencing efforts have accelerated research on flavonoid biosynthesis^10,11^. For ornamental taxa, chromosome-level assemblies of *H. syriacus*^12^ and *H. mutabilis*^13^ revealed genetic bases for floral color transition and woody growth.

Notably, *A. rosea* genomic exploration remains limited. Previous studies have conducted transcriptome sequencing on single-petaled and double-petaled red-flowered *A. rosea*, identifying several genes potentially associated with double-petaled flower formation^14,15^. Additionally, Wang *et al*.^16^. performed genome sequencing of *A. rosea* (genome data yet to be published) and resequenced 32 germplasm resources to investigate its genetic diversity. This contrasts with related genera like *Ceiba pentandra* (kapok tree) and *Bombax ceiba*, whose genomes enabled comparative analyses of fiber evolution^17^. The lack of a hollyhock nuclear genome hinders exploration of its hallmark traits: petal pigmentation diversity and mucilage biosynthesis pathways.

Despite these advances in related species, *A. rosea* genomic exploration remains limited. To address this, we present the first chromosome-level genome assembly of *A. rosea*. Combining PacBio HiFi (52x) sequencing, Hi-C scaffolding, and RNA-seq annotation, we generated a 1.01 Gbp genome with scaffold N50 of 52.57 Mbp, anchoring 92.6% (1.01 Gbp) onto 21 chromosomes. We annotated 51,436 protein-coding genes, providing a critical resource for elucidating the genetic basis of hollyhock’s ornamental and medicinal traits.

## Methods

### Sampling

The plant seed material for genome sequencing originated from wild Hollyhock seeds preserved in The Germplasm Bank of Wild Species in Southwest China, collected from Songpan, Aba, Sichuan, with the collection number SCU-10-427. Seeds were sown in plug trays during March and maintained in the nursery of Chengdu Botanical Garden (30°46′N, 104°07′E) under controlled conditions: temperature 15–20 °C, natural photoperiod, and peat-based substrate (pH 5.5–6.0). At one month post-sowing, seedlings developed approximately 3-4 true leaves, from which leaf samples were collected for preliminary genomic analysis.

Seedlings were then transplanted into 14-cm pots under adjusted conditions: temperature 20–25 °C (other parameters unchanged). By four months post-sowing, plants entered a rapid vegetative growth phase (25–35 °C), and young leaves were harvested for genome sequencing.

At 14 months post-sowing (mature flowering stage, 20–30 °C), multiple tissues (leaves, stems, fruits, flowers, and roots) from the same individual were collected for transcriptome sequencing.

### Chromosome fluorescence by *in situ* hybridization

**Fig. 2:**
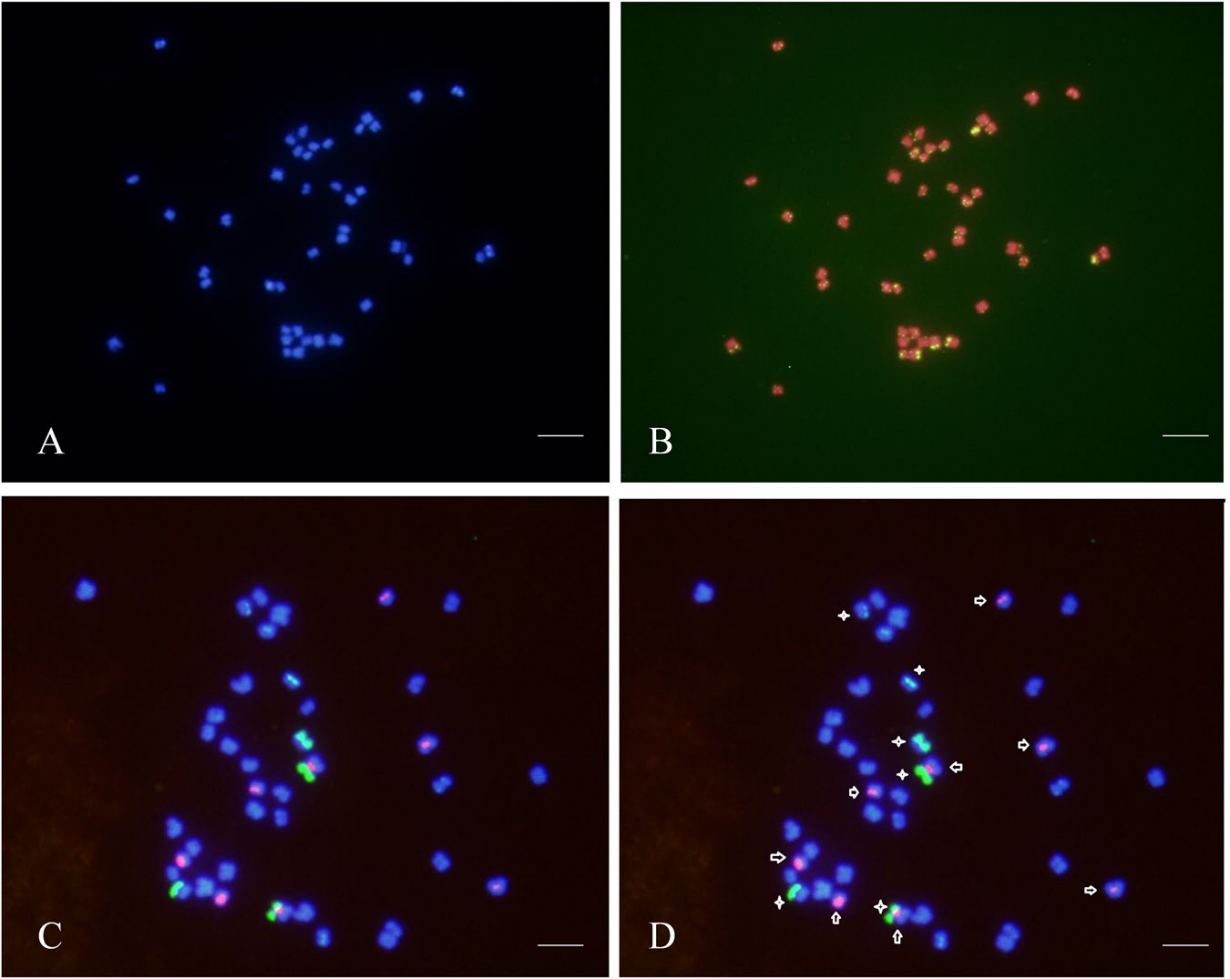
Chromosomal Fluorescence *In Situ* Hybridization (FISH) Images of *A. rosea*, AB: Telomere Fluorescence *In Situ* Hybridization (FISH) results, showing *in situ* hybridization of telomeric repeat sequences (green). CD: Chromosomal rDNA Fluorescence *In Situ* Hybridization (FISH) results, displaying 5S rDNA in red (indicated by arrows) and 18S rDNA in green (indicated by asterisks). Scale bar: 5 μm.

Take the root of *A. rosea*, which has active meristematic tissue, and use nitrous oxide to induce cell mitosis. After obtaining mid-phase cells, prepare chromosome specimens and perform fluorescence *in situ* hybridization (FISH) using fluorescent probes based on the conserved repetitive sequences of telomeres, 5SrDNA and 18SrDNA. After DAPI staining, observe chromosomes under a fluorescence microscope to determine the karyotypic features of the species. Through chromosome squashing of root tips and FISH, it was revealed that *A. rosea* has a total of 42 chromosomes, with lengths ranging from 0.8 to 2.0 μm. The majority of these chromosomes were found to be metacentric or submetacentric. Using 5S rDNA and 18S rDNA repeated sequence probes for FISH analysis, we identified 8 chromosomes with strong 5S rDNA (red) hybridization signals and 6 chromosomes with strong 18S rDNA (green) hybridization signals, confirming the diploid nature of the sample with a karyotype of 2n = 2x = 42 (Fig. 2).

### Genome size estimation

**Fig. 3:**
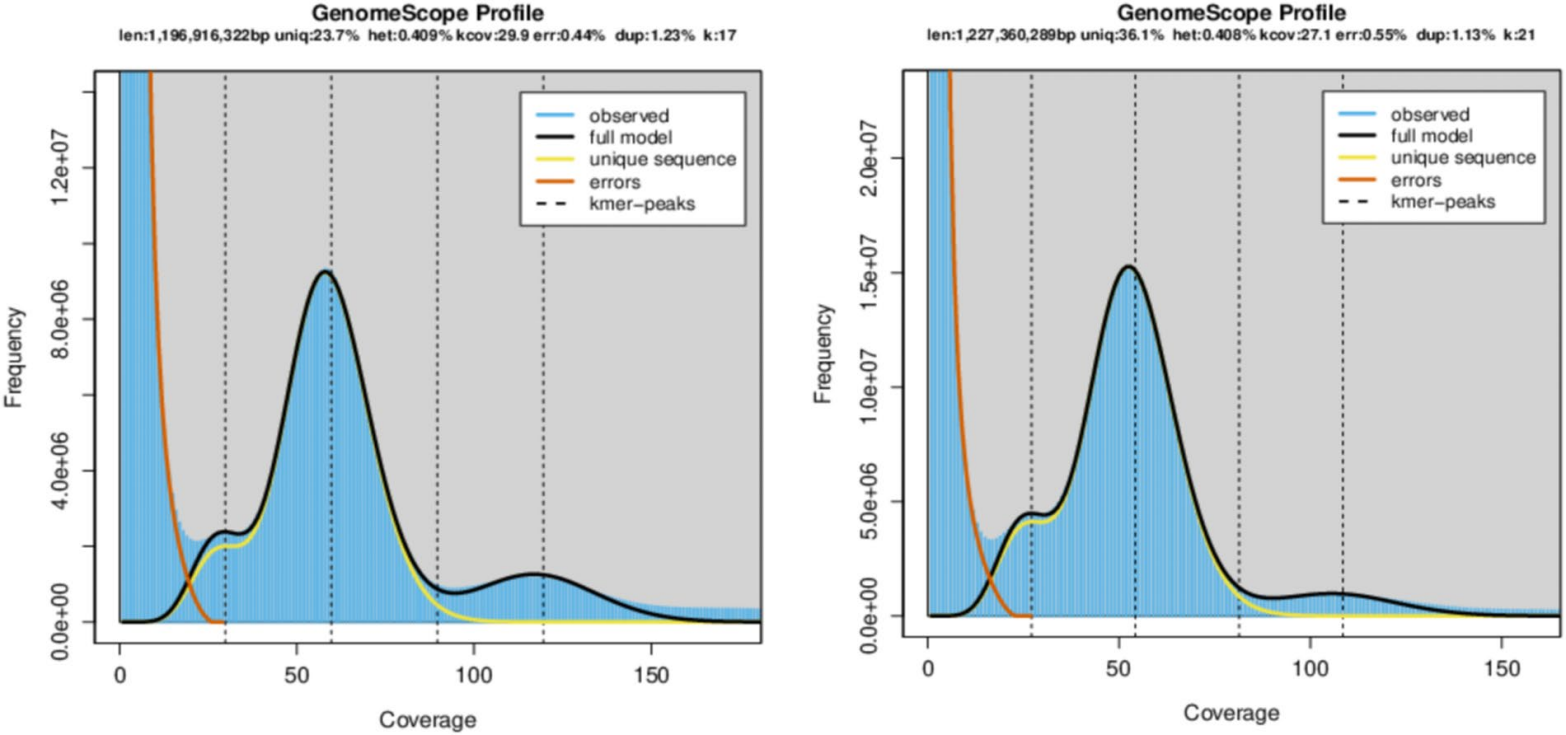
17,21-kmer Distribution Curves. Note: The x-axis represents the k-mer depth (coverage), while the y-axis denotes the frequency of k-mers at that particular depth.

To estimate the genome size of *A. rosea*, 100X short read data (PE150; DNBSEQ) performed K-mer analysis, and SOAPnuke (v 1.5.6)^18^ was used to filter the original sequencing data, and the reads with low quality, adaptor contamination and PCR duplication were filtered out. The remaining clean reads were reused for subsequent analysis. The 17,21 kmer frequency is quickly counted using the Jellyfish (v2.1.4)^19^, then GenomeScope (v1.0)^20^ is used to fit the 17,21 kmer spectrum and estimate the genome’s characteristics. The genomic survey, employing a negative binomial distribution model, estimated the species’ genome size to be around 1.2 Gbp with a heterozygosity rate of 0.41% and 76.3% repeat content, indicative of a low heterozygous, high repeat genome (Fig. 3).

### Genome sequencing

DNA was extracted using the long - fragment magnetic bead method. The procedure was as follows: Take about 0.3–3 g of tissue sample, grind it into a flour - like consistency in liquid nitrogen, and transfer it into a 5 mL centrifuge tube. Add 3 mL of Tissue Lysis Buffer 1, mix by shaking, and incubate at 65 °C for 30 minutes. After incubation, centrifuge at low temperature for 10 minutes, transfer the supernatant to a new corresponding centrifuge tube, add 1 mL of Precipitation Buffer 2, incubate at low temperature for 5 minutes, centrifuge at low temperature for 10 minutes, and transfer the supernatant to a 2 mL centrifuge tube. Add 900 µL of Lysis Supernatant to each tube, then add 900 µL of DNA Binding Buffer and 50 µL of Magnetic Bead Buffer. Gently invert and mix several times, centrifuge briefly for 2 seconds, let stand at room temperature for 5 minutes to allow binding. Place the 2 mL centrifuge tubes on a magnetic rack until the magnetic beads are adsorbed and the liquid becomes clear. Discard the supernatant, wash the magnetic bead precipitate twice with 75% ethanol, air - dry at room temperature, and add 80–300 µL of pre - warmed Elution Buffer at 50 °C. Gently tap the centrifuge tube to resuspend the magnetic beads, let stand at room temperature for 5 minutes, place on the magnetic rack until the liquid is clear, transfer the supernatant to a new 1.5 mL centrifuge tube, and store it in a refrigerator at 4 °C for nucleic acid quality control. After being disrupted by Megaruptor (approximately 15–20 K), the DNA underwent damage repair and end repair before being ligated with known adaptors. Following enzymatic digestion, selection, and final transformation into a dumbbell-shaped HiFi library, it was subjected to quality checks. Post-qualification, the sample underwent PacBio Sequel II sequencing, subsequent data analysis, and interpretation of results.

### Transcriptome sequencing

Transcriptome sequencing is used to assist in genome annotation. Total RNA was extracted from flowers, leaves, roots, stems, and fruits of the same hollyhock plant using the CTAB method^21^. mRNA was then enriched from the total RNA to construct strand-specific transcriptome libraries. These libraries were sequenced using the high-throughput DNBSEQ platform, followed by bioinformatics analysis.

### Genome assembly and Hi-C analysis

**Fig. 4:**
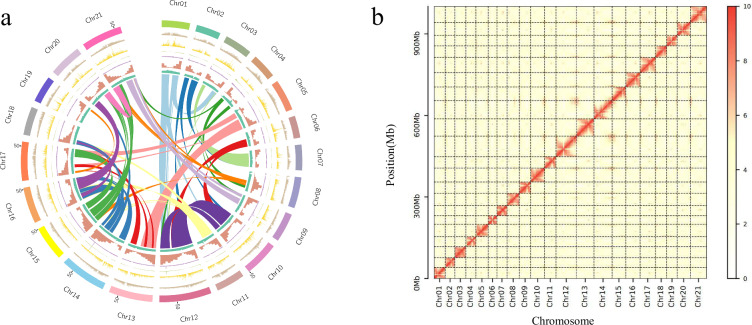
The genomic features and Hi-C map of *A.rosea*. (**a**) genomic features of *A. rosea* (From outside to inside are genomic collinearity, GC content, gene number, repeat sequence features (transposon, DNA_class.stat, Copia, gypsy) and chromosomes), (**b**) Hi-C map of the *A. rosea*, Color intensity indicates the frequency of Hi-C interaction links from low (yellow) to high (red).

The PacBio Sequel II platform generated 62.48 Gbp of HiFi long-read data (52 × coverage; 33.117 Gbp and 29.370 Gbp from two cells, respectively), which were assembled with Hifiasm (v0.19.6)^22^. The resulting contigs spanned 1.09 Gbp with an N50 of 36.61 Mbp. The genome assembly quality was assessed using BUSCO (v5.1.2)^23^, with the completeness evaluation library being eudicots_odb10. BUSCO assessment showed that the contig genome completeness was 99.5% (Table 1, Fig. 4).

**Table 1:** Statistics of the evaluation results of genome BUSCO.

Type	Number	Rate (%)
**Contig**		
Complete BUSCOs (C)	2315	99.5
Complete and single-copy BUSCOs (S)	1145	49.2
Complete and duplicated BUSCOs (D)	1170	50.3
Fragmented BUSCOs (F)	5	0.2
Missing BUSCOs (M)	6	0.3
Total BUSCO groups searched	2326	100
**Scaffold**		
Complete BUSCOs (C)	2326	99.6
Complete and single-copy BUSCOs (S)	1146	49.3
Complete and duplicated BUSCOs (D)	1170	50.3
Fragmented BUSCOs (F)	5	0.2
Missing BUSCOs (M)	5	0.2
Total BUSCO groups searched	2326	100
**Gene-set**		
Complete BUSCO	1588	98.4
Complete and single-copy BUSCOs	853	52.9
Complete and duplicated BUSCOs	735	45.5
Fragmented BUSCOs	3	0.2
Missing BUSCOs	23	1.4
Total BUSCO groups searched	1614	—

Hi-C libraries were prepared using DpnII restriction enzyme and sequenced on the DNBSEQ platform, producing 291.85 Gbp of 150 bp paired-end reads. To anchor contigs onto chromosomes, the Hi-C clean data were mapped to the assembled contigs using BWA (v0.7.12)^24^, and then erroneous mappings (MAPQ = 0) and duplicates were filtered by the Juicer^25^ pipeline (v1.5.6) to obtain the interaction matrix. Following, approximately 1.01 Gbp reads were used to anchor the contigs into chromosomes with 3D-DNA^26^ pipeline (v180,922). And 3D-DNA pipeline was used to remove select short contigs using default parameters. The Hi-C contact maps were then reviewed with Juicebox^27^ Assembly Tools (v2.15.07).

The chromosome-level genome assembly of *A. rosea* was generated by integrating PacBio HiFi long reads (62.48 Gbp, 52 × coverage) and Hi-C data (291.85 Gbp). The final assembly spans 1.01 Gbp across 21 scaffolds (scaffold N50 = 52.57 Mbp), with a contig N50 of 36.61 Mbp and a BUSCO completeness of 99.6% (eudicots_odb10) (Table 1).

### Repeat sequence annotation

The methods for repeat sequence annotation are categorized into two types: homology-based alignment and de novo prediction. Homology-based alignment relies on the RepBase^28^, RepeatMasker (v4.1.2)^29^ is used to identify and classify sequences similar to known repeat sequences. For de novo prediction, a novel repeat sequence library is first constructed using software such as RepeatModeler (v2.0.3)^30^ and LTRharvest (v1.6.2)^31^, and then predictions are made using RepeatMasker. In addition, TRF (4.10.0)^32^ is utilized to locate tandem repeat sequences within the genome. A total of 565.84 Mbp (56% of the genome) of repetitive sequences were identified (Table 2), with transposable elements (TEs) predominating, among which LTR elements accounted for 48.44% of the genome (Table 3).

**Table 2:** Statistical results of repeat repeats.

Type	Repeat Length(bp)	% of genome
Trf	118191505	11.7
Repeatmasker	150729880	14.92
Proteinmask	115731537	11.45
De novo	516766961	51.14
Total	565840497	56

**Table 3:** Classification of TEs.

Type	Length (bp)	% in genome
DNA	46813547	4.63
LINE	18109441	1.79
SINE	1129454	0.11
LTR	489490650	48.44
Other	2900	0.000287
Unknown	4892388	0.48
Total	539481100	53.39

### Gene structure prediction

Gene structure prediction integrates three approaches: homology-based prediction, de novo prediction, and transcriptome-assisted prediction. Homology-based prediction utilizes annotation information from six closely related species: *H. cannabinus*, *G. raimondii*, *G. hirsutum*, *Corchorus. capsularis*, *C. olitorius*, and *T. cacao*. Firstly, GeMoMa (v1.9)^33^ is employed for homology prediction. Based on the results of homology prediction, genes with complete structures are selected for training the de novo prediction software, Augustus (v3.0.3)^34^ and SNAP (v11/29/2013)^35^. RNA-seq data is aligned using HISAT (v2.0.4)^36^ and assembled into transcripts with StringTie (v1.2.2)^37^. Finally, EvidenceModeler (v1.1.1)^38^ is used to consolidate all data, resulting in the final gene set. The completeness and proportion of fully predicted genes are assessed by comparing the gene set against the embryophyta_odb10 database using BUSCO. Ultimately, 51,436 genes were annotated. Among these predicted genes, the average gene length and coding sequence (CDS) length were 2739.92 bp and 1242.54 bp, respectively (Table 4). The average number of exons per gene was 5.49, with average exon and intron lengths of 226.23 bp and 333.31 bp, respectively. The BUSCO completeness assessment of the gene set revealed coverage of 1,588 conserved proteins, with a completeness of 98.4%, including 853 (52.9%) single-copy genes and 735 (45.5%) multi-copy genes (Table 1).

**Table 4:** Gene structure prediction statistics.

Type	Number
Number of genes	51436
Gene length(bp)	2739.92
CDS length (bp)	1242.54
Exons per gene	5.49
Exons length (bp)	226.23
Intron length (bp)	333.31

### Functional annotation of genes

**Fig. 5:**
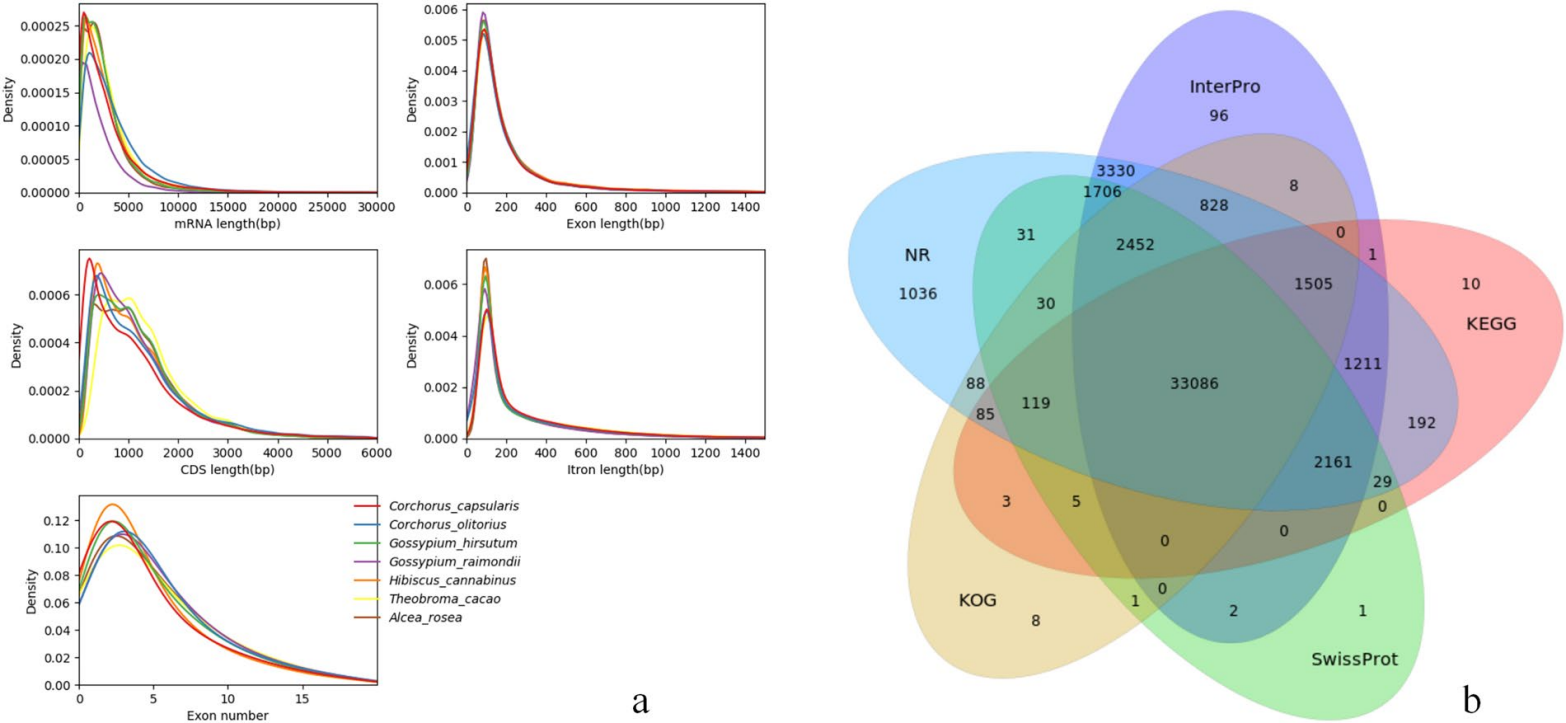
Gene structure prediction and gene function annotation. (**a**) Statistical plots of the prediction results of the gene structure, (**b**) Gene functional annotation results for number statistics in five databases: NR, InterPro, KEGG, SwissProt and KOG.

Functional annotation of genes is performed using InterProScan (v5.28-67.0)^39^ to search secondary structural domain databases and obtain gene function information. The comparison databases used include SwissProt^40^, TrEMBL, KEGG^41^, InterPro, NR, KOG, and GO^42^. The functional annotation statistics of the predicted genes in seven databases (Nr, Swissprot, KEGG, KOG, TrEMBL, Interpro, GO) are shown in the Table 5. The Venn diagram illustrating the number of gene annotations across five databases (Nr, Interpro, KEGG, Swissprot, and KOG) is shown in the Fig. 5b, with 33,086 genes annotated in all five databases. The statistical results of KOG functional annotation categorization indicated that the five most represented gene functions were ‘General function prediction only’, ‘Signal transduction mechanisms’, ‘Transcription’, ‘Function unknown’, and ‘Posttranslational modification, protein turnover, chaperones’ (Fig. S1). The KEGG pathway annotation results showed that the five most involved metabolic pathways were global and overview maps, carbohydrate metabolism, translation, folding, sorting, and degradation, and signal transduction - membrane transport (Fig. S2). The GO secondary node annotation classification statistics indicated that the most represented categories in terms of gene number were binding, cellular processes, catalytic activity, metabolic processes, and cellular component (Fig. S3).

**Table 5:** Genome function annotation result.

	Number	Percentage(%)
Total	51,436	100
Nr-Annotated	47,889	93.1
Swissprot-Annotated	39,623	77.03
KEGG-Annotated	38,407	74.67
KOG-Annotated	38,218	74.3
TrEMBL-Annotated	47,899	93.12
Interpro-Annotated	46,386	90.18
GO-Annotated	31,171	60.6
Overall	48,045	93.41

## Data Records

The genome sequence data used for genome assembly and annotation have been deposited in the Genome Sequence Archive (GSA) of the National Genomics Data Center (NGDC) under accession number CRA024326^43^. Both the raw reads and chromosome assembly have been submitted to NCBI with the accession number of SRP590903^44^ and GCA_050717195.1^45^. Annotation files can be accessed on Figshare^46^.

## Technical Validation

We assessed the genome integrity using BUSCO, using the comparison library eudicots_odb10, the assembly integrity of contig genome was 99.5%, and the assembly integrity of scaffold genome was 99.6%, and the number and proportion of fully predicted genes were evaluated. The results showed that the gene set fully covered 1,588 conserved proteins (98.4% integrity), of which 863 (52.9%) were single copies and 735 (45.5%) were multiple copies. The three evaluation results showed that the genome assembly quality of hollyhock was high.

## Supplementary information

Supplementary figure

## Data Availability

Software programs and pipelines were conducted as specified in the instruction manuals and published protocols of bioinformatic tools. Detailed information on software versions, code, and parameters can be found in the Methods section. No specific or custom code was used in this study.
