# Supplementary material for: Chromosome-level genome assembly of the ornamental plant Alcea rosea
Source: Sci Data. 2025 Jul 4;12:1145. doi: 10.1038/s41597-025-05473-z (PMC12227575; doi:10.1038/s41597-025-05473-z)
Supplement: Supplementary file 1 — Supplementary figure [file 41597_2025_5473_MOESM1_ESM.pdf]

Fig. S1 KOG Functional Enrichment Analysis.

Fig. S2 KEGG Functional Enrichment Analysis.

Fig. S3 GO Functional Enrichment Analysis.

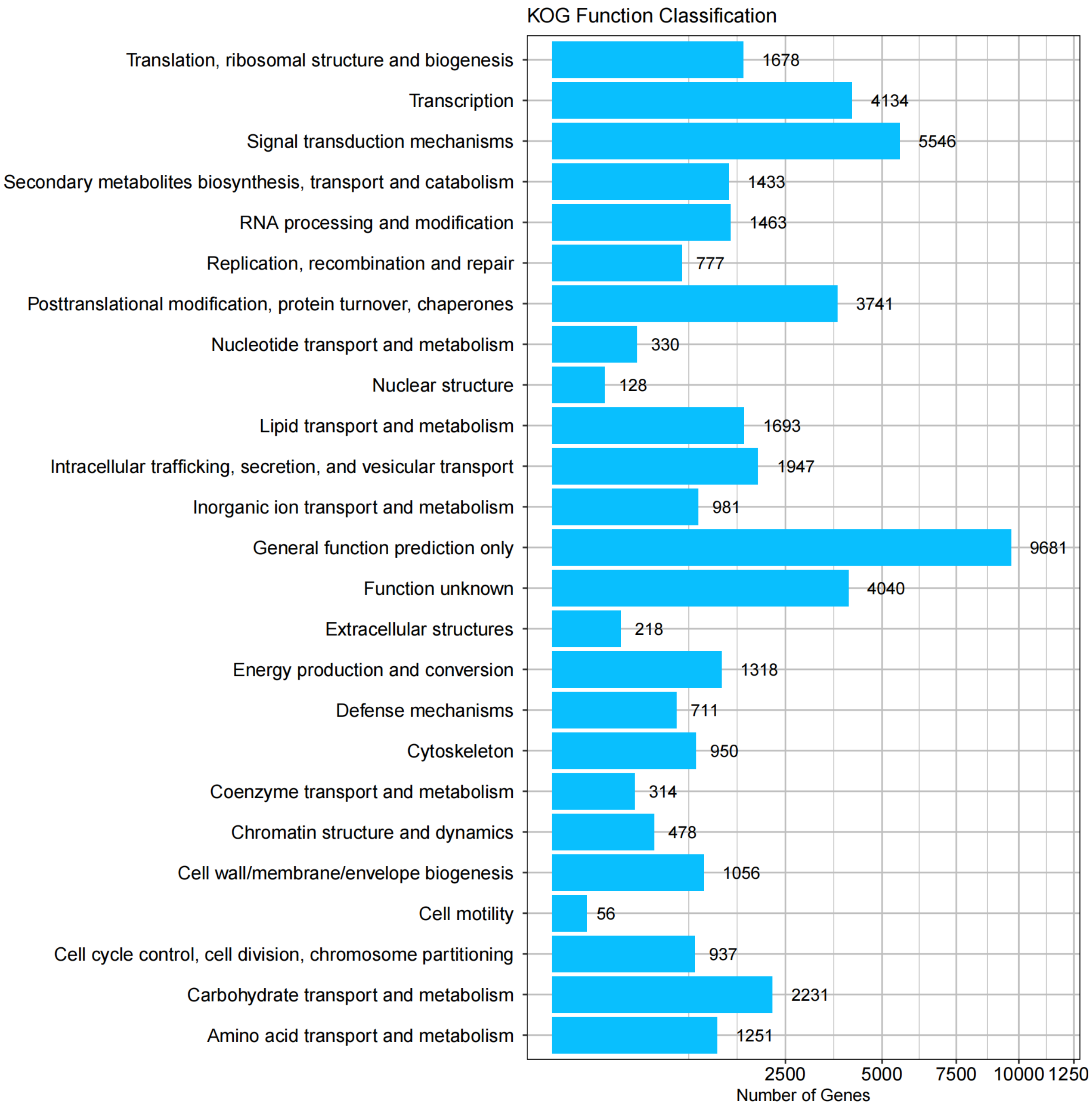

KOG Function Classification
Translation, ribosomal structure and biogenesis
1678
Transcription
4134
Signal transduction mechanisms
5546
Secondary metabolites biosynthesis, transport and catabolism
1433
RNA processing and modification
1463
Replication, recombination and repair
777
Posttranslational modification, protein turnover, chaperones
3741
Nucleotide transport and metabolism
330
Nuclear structure
128
Lipid transport and metabolism
1693
Intracellular trafficking, secretion, and vesicular transport
1947
Inorganic ion transport and metabolism
981
General function prediction only
9681
Function unknown
4040
Extracellular structures
218
Energy production and conversion
1318
Defense mechanisms
711
Cytoskeleton
950
Coenzyme transport and metabolism
314
Chromatin structure and dynamics
478
Cell wall/membrane/envelope biogenesis
1056
Cell motility
56
Cell cycle control, cell division, chromosome partitioning
937
Carbohydrate transport and metabolism
2231
Amino acid transport and metabolism
1251
2500
5000
7500
10000
1250
Number of Genes


Fig S1

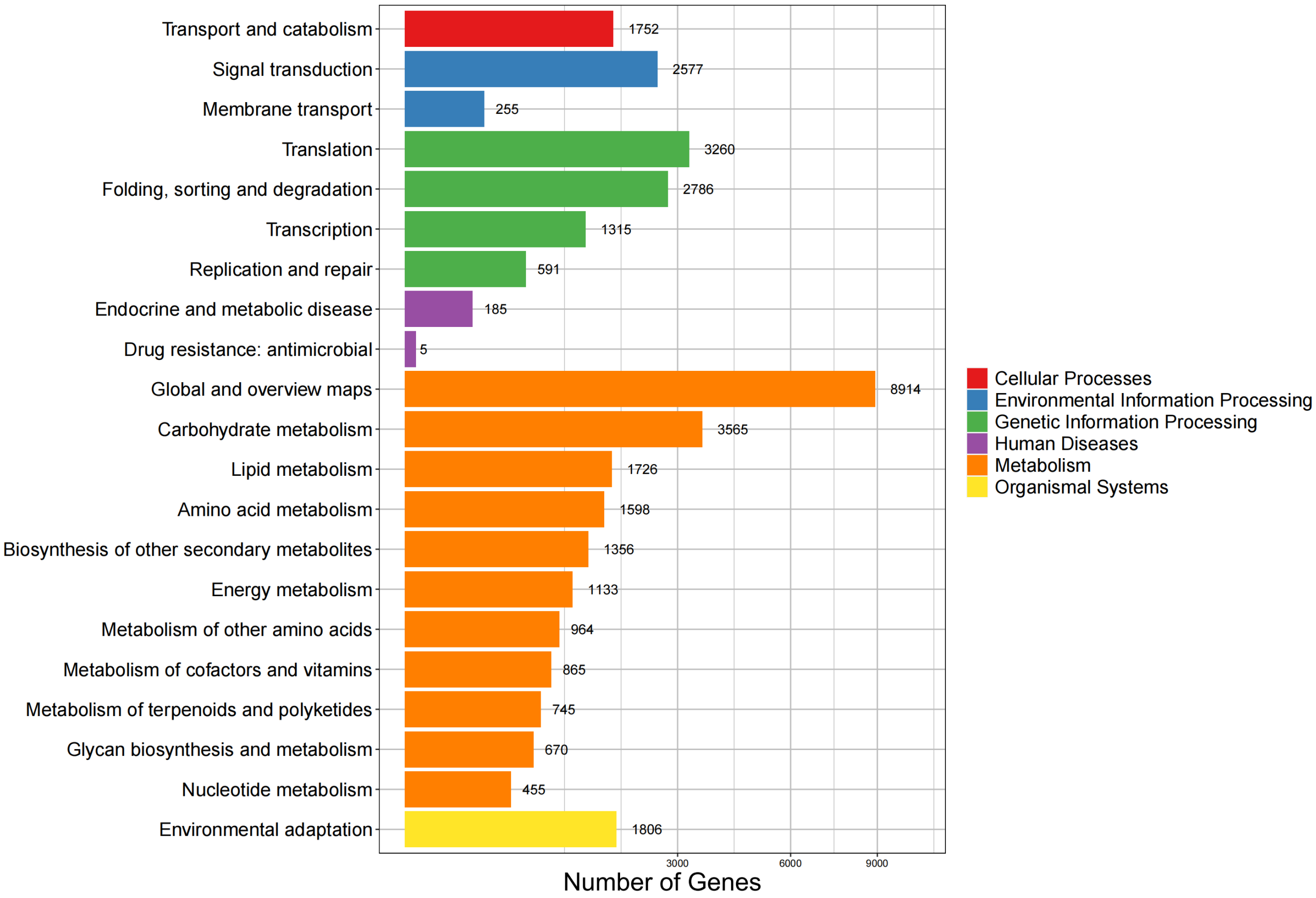
Transport and catabolism
Signal transduction
Membrane transport
Translation
Folding, sorting and degradation
Transcription
Replication and repair
Endocrine and metabolic disease
Drug resistance: antimicrobial
Global and overview maps
Carbohydrate metabolism
Lipid metabolism
Amino acid metabolism
Biosynthesis of other secondary metabolites
Energy metabolism
Metabolism of other amino acids
Metabolism of cofactors and vitamins
Metabolism of terpenoids and polyketides
Glycan biosynthesis and metabolism
Nucleotide metabolism
Environmental adaptation
1752
2577
255
3260
2786
1315
591
185
5
8914
3565
1726
1598
1356
1133
964
865
745
670
455
1806
3000
6000
9000
Number of Genes
Cellular Processes
Environmental Information Processing
Genetic Information Processing
Human Diseases
Metabolism
Organismal Systems

Fig S2

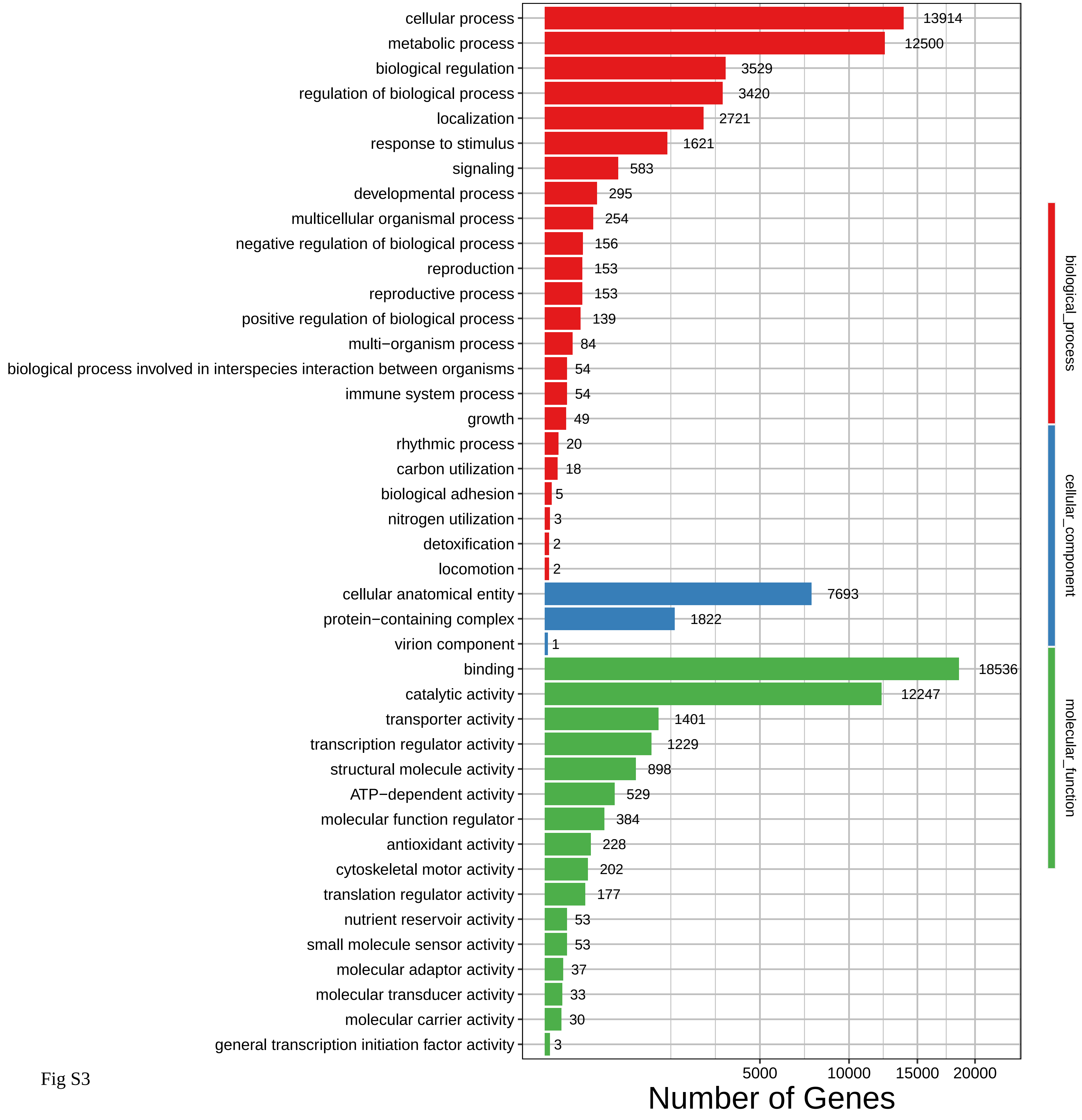
cellular process
metabolic process
biological regulation
regulation of biological process
localization
response to stimulus
signaling
developmental process
multicellular organismal process
negative regulation of biological process
reproduction
reproductive process
positive regulation of biological process
multi−organism process
biological process involved in interspecies interaction between organisms
immune system process
growth
rhythmic process
carbon utilization
biological adhesion
nitrogen utilization
detoxification
locomotion
cellular anatomical entity
protein−containing complex
virion component
binding
catalytic activity
transporter activity
transcription regulator activity
structural molecule activity
ATP−dependent activity
molecular function regulator
antioxidant activity
cytoskeletal motor activity
translation regulator activity
nutrient reservoir activity
small molecule sensor activity
molecular adaptor activity
molecular transducer activity
molecular carrier activity
general transcription initiation factor activity
13914
12500
3529
3420
2721
1621
583
295
254
156
153
153
139
84
54
54
49
20
18
5
3
2
2
7693
1822
1
18536
12247
1401
1229
898
529
384
228
202
177
53
53
37
33
30
3
5000
10000
15000
20000
Number of Genes
biological_process
cellular_component
molecular_function

Fig S3
